# Ancestry of the Iban Is Predominantly Southeast Asian: Genetic Evidence from Autosomal, Mitochondrial, and Y Chromosomes

**DOI:** 10.1371/journal.pone.0016338

**Published:** 2011-01-31

**Authors:** Tatum S. Simonson, Jinchuan Xing, Robert Barrett, Edward Jerah, Peter Loa, Yuhua Zhang, W. Scott Watkins, David J. Witherspoon, Chad D. Huff, Scott Woodward, Bryan Mowry, Lynn B. Jorde

**Affiliations:** 1 Department of Human Genetics, University of Utah, Salt Lake City, Utah, United States of America; 2 Department of Psychiatry, University of Adelaide and Royal Adelaide Hospital, Adelaide, Australia; 3 Sorenson Molecular Genealogy Foundation, Salt Lake City, Utah, United States of America; 4 Queensland Centre of Mental Health Research, Brisbane, Australia; 5 Queensland Brain Institute, University of Queensland, Brisbane, Australia; University of Glasgow, United Kingdom

## Abstract

Humans reached present-day Island Southeast Asia (ISEA) in one of the first major human migrations out of Africa. Population movements in the millennia following this initial settlement are thought to have greatly influenced the genetic makeup of current inhabitants, yet the extent attributed to different events is not clear. Recent studies suggest that south-to-north gene flow largely influenced present-day patterns of genetic variation in Southeast Asian populations and that late Pleistocene and early Holocene migrations from Southeast Asia are responsible for a substantial proportion of ISEA ancestry. Archaeological and linguistic evidence suggests that the ancestors of present-day inhabitants came mainly from north-to-south migrations from Taiwan and throughout ISEA approximately 4,000 years ago. We report a large-scale genetic analysis of human variation in the Iban population from the Malaysian state of Sarawak in northwestern Borneo, located in the center of ISEA. Genome-wide single-nucleotide polymorphism (SNP) markers analyzed here suggest that the Iban exhibit greatest genetic similarity to Indonesian and mainland Southeast Asian populations. The most common non-recombining Y (NRY) and mitochondrial (mt) DNA haplogroups present in the Iban are associated with populations of Southeast Asia. We conclude that migrations from Southeast Asia made a large contribution to Iban ancestry, although evidence of potential gene flow from Taiwan is also seen in uniparentally inherited marker data.

## Introduction

Many distinct ethnic groups reside within the Malaysian state of Sarawak, reflecting broader patterns of cultural and linguistic diversity observed throughout Island Southeast Asia (ISEA) [1]. It has been suggested that ISE Asian inhabitants descend mainly from individuals who either migrated from Southeast Asia before the Neolithic expansion from Taiwan or, alternatively, descend mainly from these Taiwanese migrants [2], [3], [4], [5]. The Iban, also referred to as Sea Dayaks, are one of the largest indigenous groups in Sarawak today [6]. They are believed to have migrated from the headwaters of the Kapuas River in the central highlands of Borneo and down into the coastal plains of present-day Sarawak in several distinct waves, the first of which took place 16 generations, approximately 400 years, ago. Comprehensive genetic analysis of the Iban will provide insight about the extent to which specific population movements influenced this population and contribute to the current understanding of ISE Asian ancestry.

**Figure 1:**
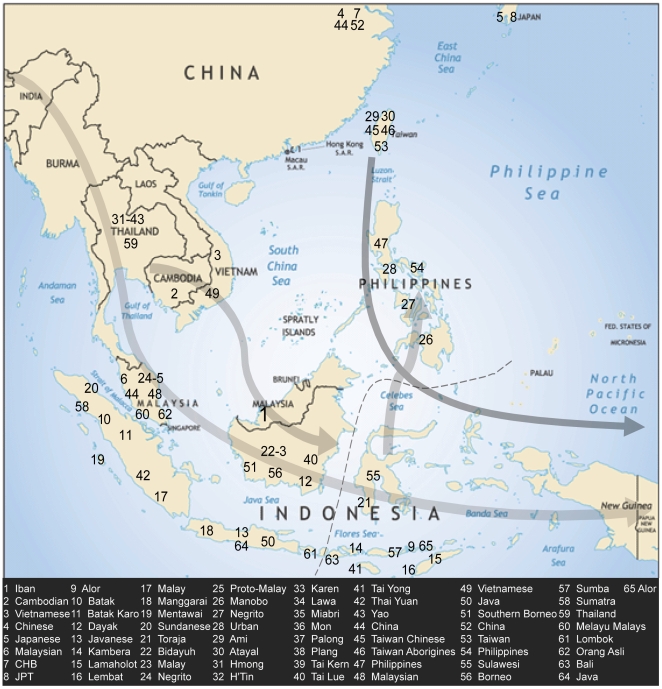
Map of ISEA and generalized migration patterns. All populations examined in this studied are assigned numbers (see Table S1). The arrows are shaded from dark to light according to consecutive migration events: 1) the Southern migration route along the Sundaland land bridge, 2) mainland Southeast Asia migrations and south-to-north migrations from Indonesia, and 3) the Neolithic gene flow from Taiwan into present-day ISEA.

It is well established that the first people to inhabit ISEA migrated across the prehistoric Sundaland land bridge that connected mainland Southeast Asia to regions as far east as Wallace's Line (Fig. 1). These early human migrations occurred approximately 45,000 to 50,000 years ago [7]. Primitive human fossil remains excavated from Niah Cave, Sarawak, where the Iban reside today, provide evidence of anatomically modern human habitation in this location at least 50,000 ago [8], [9].

Another wave of migration from mainland Southeast Asia into ISEA occurred between 12,000 and 6,000 years ago (Fig. 1) [10]. During this time, the Sunda shelf was partially flooded by rising sea level, resulting in island formation [11], [12], [13]. Humans continued to migrate into the newly formed islands during the subsequent millennia, but their genetic contribution to various ISE Asian populations is unclear. One model suggests that most of the present-day ISEA inhabitants are direct descendents of populations that migrated during this time [10], [13].

An alternative model suggests the largest ancestral contribution to ISEA populations results from a third and more recent event, which is associated with one of the largest agriculturally-driven migrations in human history: the Neolithic expansion from Taiwan. Some linguists suggest that the inhabitants who first settled in present-day ISEA, indigenous Australo-Melanesian foragers, were largely displaced by a wave of “Mongoloid” Austronesians who migrated into this region approximately 4,000 ago [14], [15]. These migrants are thought to have left South China, traveled to Taiwan, and by 4,000 ago, expanded into the Philippines and throughout ISEA and the Pacific[2], [4], [16], [17].

The maternal and paternal genetic lineages present among inhabitants throughout Taiwan, East Asia, and ISEA have been independently studied using either non-recombining Y (NRY) or mitochondrial DNA (mtDNA) polymorphism data [10], [18], [19], [20], [21]. MtDNA studies report greater diversity in Southeast Asians compared to populations in northeast Asia regions and suggest a southern origin for present-day northeastern Asians [22], [23], [24], [25], [26]. Expanding on this concept, phylogenetic mtDNA studies have been used to associate haplogroups with Southeast Asian population movements into ISEA that were driven by climate change in the later stages of the Last Glacial Maximum [10], [13], [27]. Several reports of NRY data suggest a similar pattern [18], [24], [28], [29] although influence from northern groups, such as Taiwan, is also apparent in Southeast Asians [30].

Technical advancements now provide the opportunity to address questions about ISEA history by extending beyond mtDNA and NRY analyses to that of multi-locus autosomal DNA. Recent studies of genome-wide SNP data [31] suggest a southern origin of East and Southeast Asian populations. In order to obtain a comprehensive picture of the Iban population, we analyzed marker sets for autosomal, Y, and mitochondrial chromosomes. We use this data to distinguish among various models about the origins of the Iban population of Sarawak and conclude that migrations from mainland Southeast Asia and perhaps Indonesia had the most substantial effect on present-day genetic variation in this population, and the amount of gene flow from Taiwan into ISEA is not as large as some models suggest.

## Results and Discussion

### Genome-wide autosomal variation

**Figure 2:**
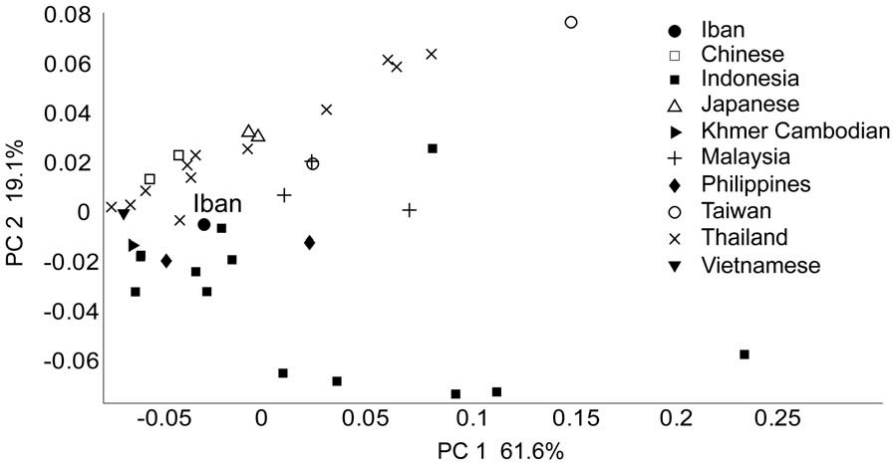
Principal Components Analysis based on genome-wide SNP genetic distances. All subpopulations are categorized into ten group as listed in Table S1. PCA coordinates for each subpopulation are provided in Table 2.

To investigate the genetic structure of the Iban and other East and Southeast Asian populations, we combined overlapping SNPs from three data sets. We genotyped approximately 250,000 genome-wide SNPs in 25 Iban individuals [32] and compared our data with Asians from the HapMap II dataset [33] and a previously published 50K SNP microarray dataset [31]. Our final dataset contains nearly 7,000 SNPs genotyped in more than 950 individuals from 39 East/Southeast Asia populations (Fig. 1; Table S1). The populations collectively represent ten different regional groups. Pairwise-population *F_ST_* values calculated between the Iban and each of the ten groups indicate that the Iban population is genetically most similar to Indonesian, Cambodian, and Thai population samples (Table 1; see Fig. S1 for admixture analysis). A principal components analysis (PCA) illustrates a similar pattern of population differentiation, with the Iban showing affinity to the mainland Southeast Asians from Thailand and also Indonesia (Fig. 2; Table 2).

**Table 1:** *F_ST_* between subpopulations in Southeast Asia.

	CHB	Chinese	Iban	Indonesia	JPT	Japanese	Cambodian	Malaysia	Philippines	Taiwan	Thailand	Vietnamese
CHB	-											
Chinese	0.002	-										
Iban	0.025	0.019	-									
Indonesia	0.020	0.015	0.012	-								
JPT	0.007	0.009	0.031	0.025	-							
Japanese	0.008	0.009	0.030	0.025	0.003	-						
Cambodian	0.012	0.009	0.013	0.006	0.019	0.020	-					
Malaysia	0.026	0.021	0.016	0.012	0.032	0.031	0.009	-				
Philippines	0.017	0.012	0.015	0.008	0.023	0.021	0.011	0.020	-			
Taiwan	0.027	0.024	0.027	0.024	0.033	0.034	0.028	0.035	0.016	-		
Thailand	0.009	0.006	0.014	0.012	0.017	0.016	0.003	0.014	0.015	0.026	-	
Vietnamese	0.006	0.002	0.015	0.010	0.015	0.016	0.006	0.015	0.009	0.024	0.003	-

**Table 2:** Sub-population PCA coordinates for Figure 2.

Population	Sub-population	PC1	PC2
Iban	Iban	−0.03	−0.01
Chinese	CHB	−0.05	0.02
	Chinese	−0.06	0.01
Indonesia	Alor	0.23	−0.06
	Batak	−0.04	−0.03
	Batak Karo	−0.02	−0.02
	Dayak	−0.02	−0.01
	Javanese	−0.06	−0.02
	Kambera	0.01	−0.07
	Lamaholot	0.09	−0.08
	Lembata	0.11	−0.07
	Malay	−0.07	−0.03
	Manggarai	0.03	−0.07
	Mentawai	0.08	0.03
	Sundanese	−0.06	−0.02
	Toraja	−0.03	−0.03
Japanese	Japanese	−0.01	0.03
	JPT	−0.01	0.03
Khmer Cambodian	Khmer Cambodian	−0.07	−0.01
Malaysia	Bidayuh	0.02	0.02
	Negrito	0.07	0.00
	Proto Malay	0.01	0.01
Philippines	Manobo	0.02	−0.01
	Urban	−0.05	−0.02
Taiwan	Ami	0.02	0.02
	Atayal	0.14	0.08
Thailand	Hmong	0.06	0.06
	H'Tin	0.08	0.06
	Karen	−0.01	0.03
	Lawa	0.03	0.04
	Mon	−0.04	0.00
	Palong	0.06	0.06
	Plang	−0.04	0.01
	Tai Kern	−0.06	0.01
	Tai Lue	−0.04	0.02
	Tai Yong	−0.07	0.00
	Thai Yuan	−0.08	0.00
	Yao	−0.04	0.02
Vietnamese	Vietnamese	−0.07	0.00

In addition to SNP analyses, we assayed 45 short tandem repeats (STRs) in the Iban, Chinese, Japanese, and a group of Southeast Asians comprised of Cambodian, Vietnamese, and Malaysian individuals as previously described (Table S2) [34], [35], [36]. Patterns of genetic differentiation (*R_ST_*) based on these data matched those observed among comparable samples using the SNP data. The shortest genetic distance observed is between the Iban and peninsular Southeast Asians (Table S7). The Malaysian and Cambodian populations and the Japanese and Chinese populations exhibit the greatest and least genetic affinity to the Iban, respectively.

These analyses indicate that the Iban are most similar to populations located in mainland Southeast Asia and Indonesia (Table 1; Table S7), suggesting that the genetic contribution of Taiwanese populations is minor. These results are inconsistent with the hypothesis that Taiwanese groups nearly replaced the populations indigenous to ISEA during the Neolithic expansion.

### NRY chromosome haplogroups

**Figure 3:**
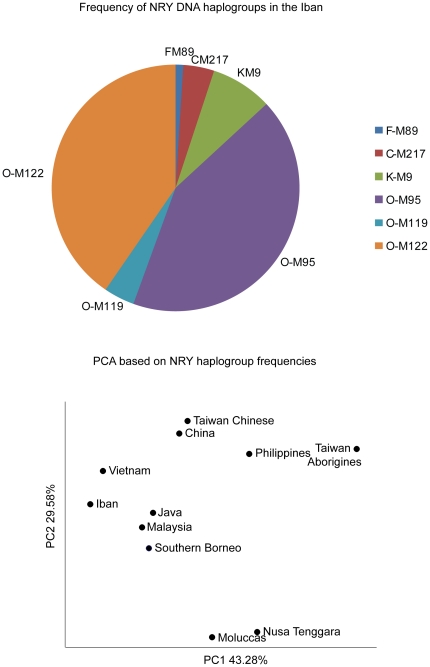
NRY chromosome haplogroup frequencies in the Iban in relation to other Asian populations.

Uniparental marker analyses also indicate a strong genetic influence from mainland Southeast Asia, although there is substantial influence from paternal lineages appears to be associated with northern Asian groups (Table S2; Table S3). The NYR haplogroup frequencies in the Iban and their relation to other populations are shown in Fig. 3. PC1 separates the Taiwanese Aborigines, Philippines, Nusa Tenggara, and Moluccas from the Iban, other Southeast Asian populations, and the Chinese populations. The separation between the Iban and an aboriginal Taiwanese group based on PC1 argues against strong Taiwanese influence on the Iban. On PC2, the Iban, Vietnamese, Chinese, Philippines, and Aboriginal Taiwanese cluster separately from the Malaysian, Southern Bornean, and to the greatest extreme, the Nusa Tenggara and Moluccas populations.

Among the 89 Iban males, the NRY O sub-haplogroups (frequency of O2a  =  0.42 and O3  =  0.40) are the most frequent. Haplogroup O2a is found at high frequency throughout Southeast Asia and is common among indigenous, isolated populations such as the Hainan Aborigines located off the mainland coast of Southeast Asia [24]. These results suggest a similar prehistory in the Iban and these Southeast Asian populations. The next most frequent NRY haplogroup is O3, which is distributed throughout East Asia, ISEA, and Oceania, and may represent a substantial contribution from Taiwan [18]. Haplogroups O1, K, C, and F are also present, but at lower frequencies (0.04, 0.08, 0.04, and 0.01, respectively). Haplogroup O1 may reflect the impact of the Out-of-Taiwan migration, although better resolution is necessary to specify Taiwan as the source population. The K, C, and F haplogroups are thought to have originated in Melanesian, Asian, and out-of-Africa migrant populations, respectively. The NRY haplogroup frequencies reflect male-specific gene flow from Southeast Asia, although they do not preclude more recent but less substantial contributions from northern populations such as that of Taiwan.

### MtDNA haplotypes

**Figure 4:**
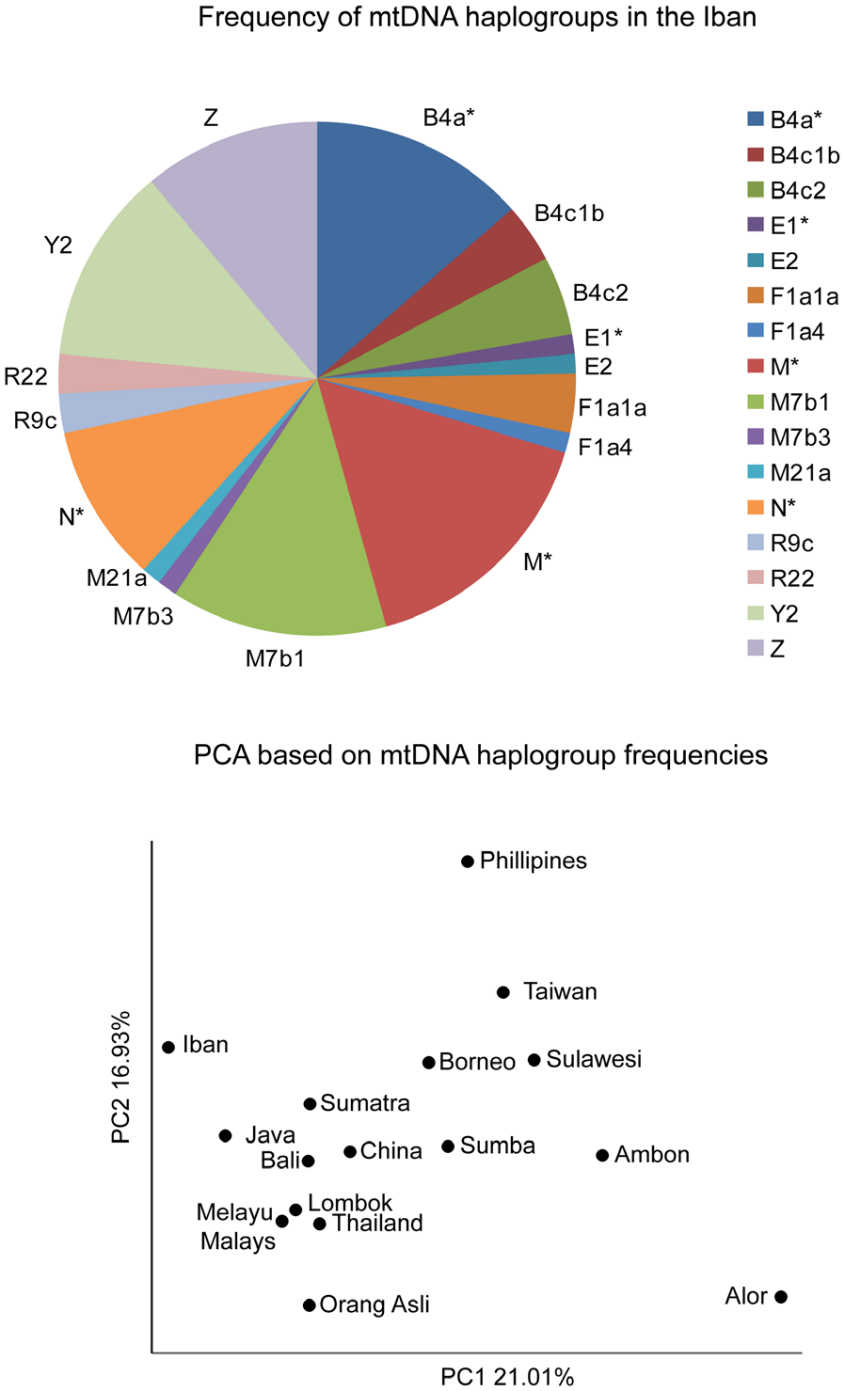
MtDNA haplogroup frequencies in the Iban in relation to other Asian populations.

In a PCA of the mtDNA haplogroup frequencies, the Iban and southwestern populations (from Sumatra, Java, Bali, Lombok, Melayu Malay, Thailand, and Orang Asli) are separated from all other populations on PC1 (Fig. 4). PC2 separates the Iban from Philippine, Taiwan, Sulawesi, Ambon, Sumba, and other groups from Borneo.

A previous study [10] provides age estimates for the mtDNA haplogroups studied here, and these estimates have been correlated with the three major human migrations in ISEA discussed above. We identified sixteen haplogroups associated with each of these categories among 83 Iban individuals (Table S4; Table S5). The haplotypes in the Iban samples reflect signatures of indigenous, late-Pleistocene, and Neolithic migrations throughout Asia [10], although age estimates must be interpreted with caution [37].

The most common haplogroup among the Iban is M* (16%), which appears to represent ancient lineages within ISEA [10]. Other observed haplogroups thought to originate >25,000 ago include: R22, found among individuals from ISEA, mainland Southeast Asia, and the Nicobar Islands [38]; R9c, most frequent in the Alor population east of Wallace's line; and M21a, notably most common among the Orang Asli [10]. Haplogroup Z, which is found in China, Mainland SEA, Sumatra, and other populations from Borneo is present at 11.11% in the Iban. These results indicate that various ancient mtDNA haplogroups thought to be associated with the first migrations into ISEA are present in the Iban.

Several lineages are also associated with prehistoric migrations during the Last Glacial Maximum, a second major migration wave, when coastlines within the ISEA region nearly doubled in length and approximately half of Sundaland was covered by water [39], [40]. Nearly one-fourth of Iban mtDNA haplogroups may originate from this migration event, supporting the hypothesis that environmental factors, specifically climate change and post-glacial flooding, influenced the demographic history of this population [10], [13]. This is largely supported by the second most common haplogroup identified among the Iban, B4a* (13.85%), which dates to the late Pleistocene in ISEA [10]. B4c2 is found at considerable frequency (4.9%) and is considered a “relict” haplogroup, dating to 13,000 ago in ISEA. Two additional low-frequency haplogroups of interest that also fall within this time frame include E1 and E2. These subclades originated at 17,000 ago and 9,500 ago, respectively, and are thought to stem from northeast Sundaland or northwest Wallacea, the present-day Indonesian islands east of Borneo [13]. The remaining haplotypes associated with the late-Pleistocene and early-Holocene migrations include M7b3, found in Taiwan and ISEA, and F1a1a, which is common throughout western and southern ISEA and Thailand and is present among aboriginal groups of the mainland Southeast Asia peninsula [41].

The previously described mtDNA haplogroups associated with the Neolithic Taiwan expansion [2], [10] found in our Iban sample include the Y2 (12.35%)and F1a4 (1.23%) lineages. The combined frequency of these haplogroups is less than that associated with migration events that occurred prior to this population movement.

The results presented in this study, which are based on both autosomal and uni-parentally transmitted markers, highlight the unique genetic history of the Iban people of Sarawak. Analyses of autosomal data indicate that the Iban are most similar to mainland Southeast Asian groups and suggest that gene flow from Taiwanese agriculturalists appears to be relatively minor in contrast to that from mainland Southeast Asians and Indonesians. The results of NRY and mtDNA haplogroup analyses complement the autosomal analyses by suggesting less gene flow from the agriculturalist expansion from Taiwan than has been previously claimed for ISEA populations [17]. The majority of mtDNA haplogroups and the greatest proportion of NRY lineages identified in our Iban sample are associated with population movements that occurred prior to this expansion. More NRY haplogroups than mtDNA haplogroups were introduced into this population during the Neolithic expansion, but the proportion of NRY haplogroups attributed to this more recent event is still less than half of the total NRY haplogroups identified. Therefore, it appears that migrations during the Neolithic did not eradicate pre-Neolithic groups. Additional sampling of indigenous ISEA populations like the Iban, in addition to genome-wide and model-based analyses, will help to further clarify the population history of this region.

## Methods

### Data collection

We collected DNA samples for 94 unrelated Iban individuals from Sarawak. Since the Iban is traditionally a preliterate society, with some community elders unable to read or write, informed consent was obtained verbally and recorded on videotape. This procedure was approved by local institutional ethics committees (Sarawak Department of Health; the University of Malaysia, Sarawak; Department of Psychiatry, University of Adelaide, Adelaide; Queensland Centre for Mental Health Research, Brisbane, Australia; University of Queensland, Brisbane, Australia) [8], [32], [42], [43]. We compared autosomal, Y-chromosome, and mtDNA SNP and sequence data to previously reported and publicly available data sets (Table S2). The populations and data sets are shown in Fig. 1 and Table 2.

### Autosomal Genotyping and Analyses

We used Affymetrix Nsp1 technology to survey ∼250,000 single nucleotide polymorphisms (SNPs) across the genomes of 25 Iban individuals [32]. Using default parameters for the Birdseed algorithm (version 2), we determined genotypes for all samples and analyzed genotypic data using the Affymetrix Genotyping Console 3.1 (Affymetrix, Santa Clara, CA, USA). We compared these data with ∼7,000 overlapping SNPs previously genotyped by the HUGO Pan-Asian SNP Consortium (HUGO) using the Affymetrix 50K Xba platform (Table S1) [31]. In order to determine patterns of variation in the genome-wide SNP data, we calculated a population pairwise *F_ST_* genetic distance and performed principal components analysis (PCA) based on these genetic distances as previously described [32]. SNP heterozygosity for the Iban and other Asian populations is provided in Table S6.

To obtain STR genotypes, we combined PCR amplicons in a multiplex reaction comprised of five to ten markers on the Applied Biosystems 3100 Genetic Analyzer. Genotype calls were based upon fluorescence signal and size per ABI GS500-LIZ size standard. We calculated genetic distance estimates (*R_ST_*) using STR data for the Iban, Chinese, Japanese, and Southeast Asians with the ARLEQUIN 3.1 software package [44].

### Non-recombining Y chromosome (NRY) and mitochondrial (mt) DNA genotyping and analyses

We assayed NRY chromosome haplogroup information using 27 Y-chromosome haplogroup/lineage-defining markers and mtDNA haplogroups using 45 mitochondrial coding region SNPs ascertained in populations from ISEA and surrounding regions [10], [45], [46], [47], [48], [49] (Table S2). The marker combinations used to determine mtDNA haplogroup/lineages are listed in Table S7. We analyzed PCR amplicons containing NRY and mtDNA haplotype and lineage-defining SNP regions on the Applied Biosystems 3100 using single-base extension SNaPshot chemistry in multiplex reactions of five to eight markers. We supplemented the haplogroup/lineage-defining coding region mtDNA SNPs with hypervariable sequence 1 polymorphisms (HVS1 sequence from position 16,000 to 16,411) obtained with BigDye 3.1 dye-terminator fluorescent sequencing (see Table S8 for estimates of nucleotide diversity).

We compared Iban Y chromosome and mtDNA haplogroup frequencies to Y chromosome haplogroup frequencies from populations in China, Taiwan Chinese, Taiwan Aborigine, Philippines, Vietnam, Malaysia, Java, Southern Borneo, Moluccas, and Nusa Tenggara males (data from [50]) and mtDNA haplogroup frequency data from throughout ISEA [10]. PCA plots constructed using haplogroup frequencies were generated using MatLab (ver. r2008).

## Supporting Information

Figure S1 ADMIXTURE analysis of the Iban and East Asian populations.
(TIF)

Table S1 List of populations and corresponding data used for analyses.
(DOCX)

Table S2 List of STR, NRY chromosome, and mtDNA markers analyzed in this study. SNPs overlapping the HGDP data set and the Affymetrix 6.0 chip used for our samples are described in Xing et al. 2009.
(DOCX)

Table S3 NRY haplogroup frequencies in the Iban compared to previously reported population frequencies (Kayser et al. 2003).
(DOCX)

Table S4 MtDNA haplogroup frequencies in the Iban and neighboring populations (Hill et al. 2007).
(DOCX)

Table S5 MtDNA haplogroup definitions for the Iban population.
(DOCX)

Table S6 SNP heterozygosity.
(DOCX)

Table S7 *R_ST_*estimates based on STR analysis.
(DOCX)

Table S8 Nucleotide diversity estimates for HVS-1 mtDNA.
(DOCX)
