# Supplementary figures and images for: Ancestry of the Iban Is Predominantly Southeast Asian: Genetic Evidence from Autosomal, Mitochondrial, and Y Chromosomes
Source: PLoS One. 2011 Jan 31;6(1):e16338. doi: 10.1371/journal.pone.0016338 (PMC3031551; doi:10.1371/journal.pone.0016338)

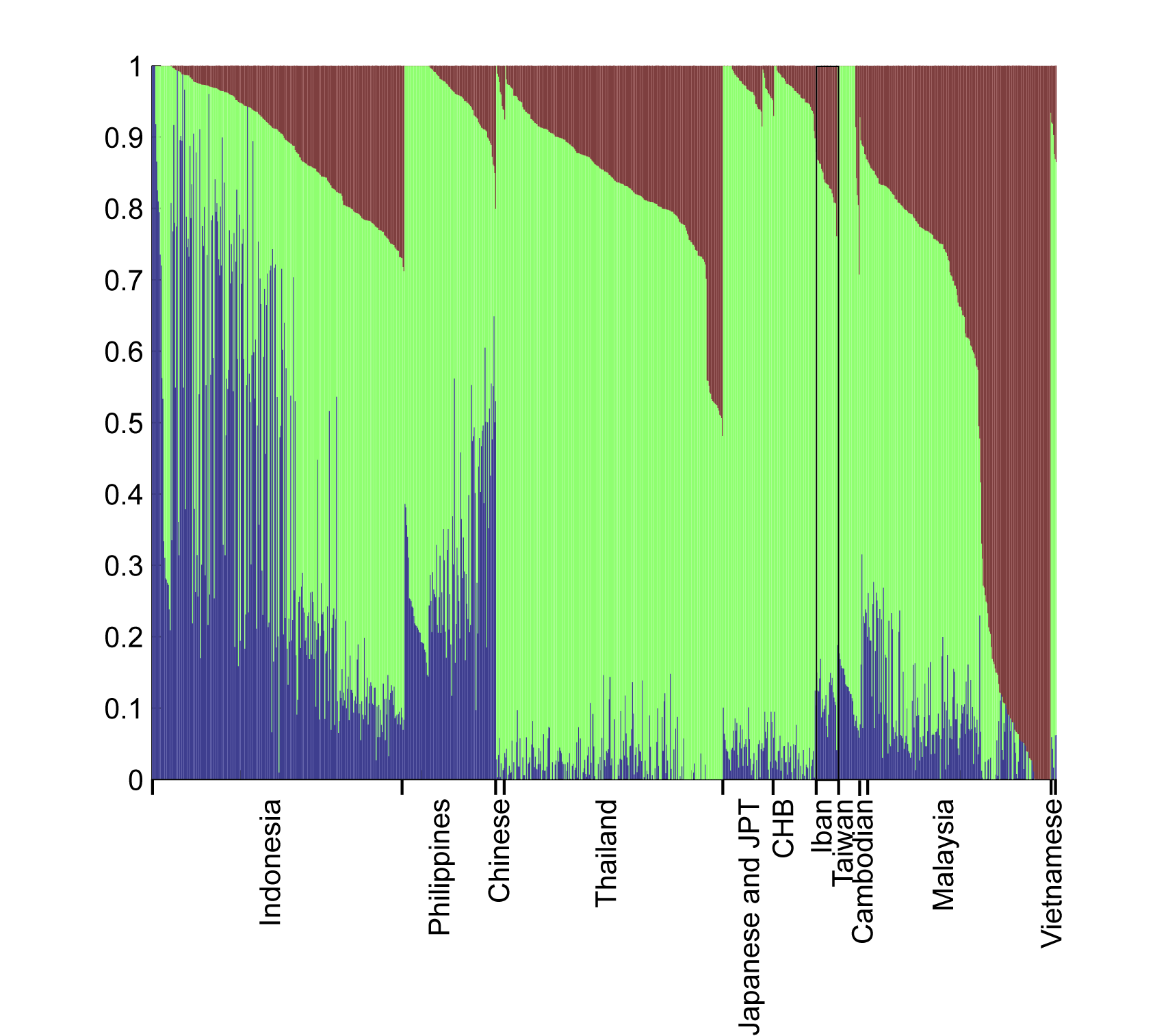

Supplement: Figure S1 — ADMIXTURE analysis of the Iban and East Asian populations. (TIF) [file pone.0016338.s001.tif]
