# Supplementary material for: Ancestry of the Iban Is Predominantly Southeast Asian: Genetic Evidence from Autosomal, Mitochondrial, and Y Chromosomes
Source: PLoS One. 2011 Jan 31;6(1):e16338. doi: 10.1371/journal.pone.0016338 (PMC3031551; doi:10.1371/journal.pone.0016338)
Supplement: Table S1 — List of populations and corresponding data used for analyses. (DOCX) [file pone.0016338.s002.docx]

Table S1. List of populations and corresponding data used for analyses

| **Reference** |  | **Population** | **Population Assignment** | **Sample Size** | **~7,000 overlapping SNP data points** | **45 STR Loci** | **NRY** | **Mt DNA** |
| --- | --- | --- | --- | --- | --- | --- | --- | --- |
|  | **Population Designation for SNP analyses** |  |  |  |  |  |  |  |
| **Jorde Samples** | Iban | Iban | 1 | 94 | **25** | **94** | **89** | **83** |
|  | Cambodian | Cambodian | 2 | 12 | **X** | **X** | **X** | **X** |
|  | Vietnamese | Vietnamese | 3 | 9 | **X** | **X** | **X** | **X** |
|  | Chinese | Chinese | 4 | 10 | **X** | **X** | **X** | **X** |
|  | Japanese | Japanese | 5 | 13 | **X** | **X** | **X** | **X** |
|  |  | Malaysian | 6 | 5 |  | **X** | **X** | **X** |
| **HapMap Phase 2** | Chinese | CHB | 7 | 45 | **X** |  |  |  |
|  | Japanese | JPT | 8 | 45 | **X** |  |  |  |
| **HUGO** | Indonesia | Alor | 9 | 19 | **X** |  |  |  |
|  |  | Batak | 10 | 20 | **X** |  |  |  |
|  |  | BatakKaro | 11 | 17 | **X** |  |  |  |
|  |  | Dayak | 12 | 12 | **X** |  |  |  |
|  |  | Javanese | 13 | 53 | **X** |  |  |  |
|  |  | Kambera | 14 | 20 | **X** |  |  |  |
|  |  | Lamaholot | 15 | 20 | **X** |  |  |  |
|  |  | Lembata | 16 | 19 | **X** |  |  |  |
|  |  | Malay | 17 | 12 | **X** |  |  |  |
|  |  | Manggarai | 18 | 36 | **X** |  |  |  |
|  |  | Mentawai | 19 | 15 | **X** |  |  |  |
|  |  | Sundanese | 20 | 25 | **X** |  |  |  |
|  |  | Toraja | 21 | 20 | **X** |  |  |  |
|  | Malaysia | Bidayuh | 22 | 50 | **X** |  |  |  |
|  |  | Malay | 23 | 38 | **X** |  |  |  |
|  |  | Negrito | 24 | 80 | **X** |  |  |  |
|  |  | Proto-Malay | 25 | 49 | **X** |  |  |  |
|  | Philippines | Manobo | 26 | 18 | **X** |  |  |  |
|  |  | Negrito | 27 | 67 | **X** |  |  |  |
|  |  | Urban | 28 | 59 | **X** |  |  |  |
|  | Taiwan | Ami | 29 | 10 | **X** |  |  |  |
|  |  | Atayal | 30 | 10 | **X** |  |  |  |
|  | Thailand | Hmong | 31 | 20 | **X** |  |  |  |
|  |  | H'Tin | 32 | 18 | **X** |  |  |  |
|  |  | Karen | 33 | 20 | **X** |  |  |  |
|  |  | Lawa | 34 | 19 | **X** |  |  |  |
|  |  | Miabri | 35 | 18 | **X** |  |  |  |
|  |  | Mon | 36 | 19 | **X** |  |  |  |
|  |  | Palong | 37 | 18 | **X** |  |  |  |
|  |  | Plang | 38 | 18 | **X** |  |  |  |
|  |  | Tai Kern | 39 | 18 | **X** |  |  |  |
|  |  | Tai Lue | 40 | 20 | **X** |  |  |  |
|  |  | Tai Yong | 41 | 18 | **X** |  |  |  |
|  |  | Thai Yuan | 42 | 20 | **X** |  |  |  |
|  |  | Yao | 43 | 19 | **X** |  |  |  |
| **Kayser et al. 2003** |  | China | 44 |  |  |  | **X** |  |
|  |  | Taiwan Chinese | 45 |  |  |  | **X** |  |
|  |  | Taiwan Aborigines | 46 |  |  |  | **X** |  |
|  |  | Philippines | 47 |  |  |  | **X** |  |
|  |  | Malaysian | 48 |  |  |  | **X** |  |
|  |  | Vietnamese | 49 |  |  |  | **X** |  |
|  |  | Java | 50 |  |  |  | **X** |  |
|  |  | Southern Borneo | 51 |  |  |  | **X** |  |
| **Hill et al. 2007** |  | China | 52 |  |  |  |  | **X** |
|  |  | Taiwan | 53 |  |  |  |  | **X** |
|  |  | Philippines | 54 |  |  |  |  | **X** |
|  |  | Sulawesi | 55 |  |  |  |  | **X** |
|  |  | Borneo | 56 |  |  |  |  | **X** |
|  |  | Sumba | 57 |  |  |  |  | **X** |
|  |  | Sumatra | 58 |  |  |  |  | **X** |
|  |  | Thailand | 59 |  |  |  |  | **X** |
|  |  | Melayu Malays | 60 |  |  |  |  | **X** |
|  |  | Lombok | 61 |  |  |  |  | **X** |
|  |  | Orang Asli | 62 |  |  |  |  | **X** |
|  |  | Bali | 63 |  |  |  |  | **X** |
|  |  | Java | 64 |  |  |  |  | **X** |
|  |  | Alor | 65 |  |  |  |  | **X** |
