# Supplementary material for: Ancestry of the Iban Is Predominantly Southeast Asian: Genetic Evidence from Autosomal, Mitochondrial, and Y Chromosomes
Source: PLoS One. 2011 Jan 31;6(1):e16338. doi: 10.1371/journal.pone.0016338 (PMC3031551; doi:10.1371/journal.pone.0016338)
Supplement: Table S2 — List of STR, NRY chromosome, and mtDNA markers analyzed in this study. SNPs overlapping the HGDP data set and the Affymetrix 6.0 chip used for our samples are described in Xing et al. 2009. (DOCX) [file pone.0016338.s003.docx]

Table S2. List of STR, NRY chromosome, and mtDNA markers analyzed in this study. SNPs overlapping the HGDP data set and the Affymetrix 6.0 chip used for our samples are described in Xing et al. 2009

| **STR** | UT1091 | UT1201 | UT1205 | UT1220 | UT1227 |
| --- | --- | --- | --- | --- | --- |
|  | UT1228 | UT1232 | UT1239 | UT1243 | UT1257 |
|  | UT1313 | UT1352 | UT1357 | UT1376 | UT1674 |
|  | UT1708 | UT1740 | UT1747 | UT1880 | UT1885 |
|  | UT1917 | UT1950 | UT1985 | UT2021 | UT2081 |
|  | UT2092 | UT2203 | UT2127 | UT5022 | UT5027 |
|  | UT5029 | UT5030 | UT5033 | UT5048 | UT5492 |
|  | UT6507 | UT6516 | UT6540 | UT7131 | UT8067 |
|  | UT868 | UT871 | UT901 | UT919 | vWFII |
| **NRY** | C-M216 | F/R-M89 | G-M201 | H-M52 | I-M170 |
|  | J2-M172 | J-P12f2 | K*-M9 | K1-SRY9138 | K2-M70 |
|  | L-M20 | M-M5 | N-LLY22g | O-M175 | O1-M119 |
|  | O2a1-M88 | O2a-M95 | O2b-SRY465 | O2-P31 | O3-M122 |
|  | P*-M74 | Q3-M3 | Q-P36 | R*-M207 | R1a-SRY10831.2 |
|  | R1-M173 | R2-M124 |  |  |  |
| **MtDNA** | C-A13263G | D-C4883T | D-C5178A | E1-G10834A | E-G7598A |
|  | E-G7598A | F-G10310A | G-A4833G | G-A4833G | H/V-T14766C |
|  | J-A12612G | K/U8-G9055A | L2-C10810T | M10-G709A | M12G-G14569A |
|  | M21-T11482C | M29Q-T13500C | M7bc-T199C | M7-C6455T | M9-C4491T |
|  | M-C10400T | M-G15043A | N9-C5417T | N-A15301G | N-C10873T |
|  | N-C10873T | N-C10873T | N-G8701A | Q3-C4335T | Q-T4117C |
|  | R-T12705C | T-T10463C | U-A12308G | U2-A3720G | U2-K-A1811G |
|  | U3-G9266A | U4-T4646C | U5a/b-A7768G | U5a1-A14793G | U6-A3348G |
|  | U7-C5360T | W-G8994A | Y-A7933G | Z-T152C | Z-T9090C |
