# Supplementary material for: Ancestry of the Iban Is Predominantly Southeast Asian: Genetic Evidence from Autosomal, Mitochondrial, and Y Chromosomes
Source: PLoS One. 2011 Jan 31;6(1):e16338. doi: 10.1371/journal.pone.0016338 (PMC3031551; doi:10.1371/journal.pone.0016338)
Supplement: Table S3 — NRY haplogroup frequencies in the Iban compared to previously reported population frequencies (Kayser et al. 2003). (DOCX) [file pone.0016338.s004.docx]

Table S3. NRY haplogroup frequencies in the Iban compared to previously reported population frequencies (Kayser et al. 2003)

|  | F-M89 | C-RPS4Y711 | C-M217 | C-M38 | C-M208 | C-390.1del | K-M9 | K-M230 |
| --- | --- | --- | --- | --- | --- | --- | --- | --- |
| **Iban** | **1** |  | **4** |  |  |  | **8** |  |
| China |  |  | 5.6 |  |  |  | 11.1 |  |
| Taiwan Chinese |  |  | 3.8 |  |  |  | 3.8 |  |
| Taiwan Aborigines |  |  |  |  |  |  |  |  |
| Philippines | 2.6 | 10.3 |  |  |  |  | 2.6 |  |
| Vietnam |  |  | 9.1 |  |  |  |  |  |
| Malaysia | 5.6 |  | 11.1 |  |  |  | 11.1 |  |
| Java | 1.9 | 1.9 |  |  |  |  | 1.9 |  |
| Southern Borneo | 5 |  | 2.5 | 2.5 |  |  | 10 |  |

| M-M4 | M-M104 | O-M175 | O-M95 | O-M119 | O-M122 | P-M74 | R-M173 |
| --- | --- | --- | --- | --- | --- | --- | --- |
|  |  |  | **42** | **4** | **40** |  |  |
|  |  |  | 2.8 | 22.2 | 58.3 |  |  |
|  |  | 11.5 |  | 23.1 | 57.7 |  |  |
|  |  | 4.7 | 4.7 | 79.1 | 11.6 |  |  |
|  |  | 2.6 | 2.6 | 41 | 35.9 |  | 2.6 |
|  |  |  | 36.4 | 9.1 | 45.5 |  |  |
|  |  |  | 27.8 | 11.1 | 27.8 |  | 5.6 |
|  |  | 1.9 | 41.5 | 22.6 | 22.6 | 1.9 | 3.8 |
|  |  | 5 | 37.5 | 15 | 17.5 | 2.5 | 2.5 |
