# Supplementary material for: Ancestry of the Iban Is Predominantly Southeast Asian: Genetic Evidence from Autosomal, Mitochondrial, and Y Chromosomes
Source: PLoS One. 2011 Jan 31;6(1):e16338. doi: 10.1371/journal.pone.0016338 (PMC3031551; doi:10.1371/journal.pone.0016338)
Supplement: Table S4 — MtDNA haplogroup frequencies in the Iban and neighboring populations (Hill et al. 2007). (DOCX) [file pone.0016338.s005.docx]

Table S4. MtDNA haplogroup frequencies in the Iban and neighboring populations (Hill et al. 2007)

| **Haplogroup** | B* | B4* | B4a* | B4a1a1 | B4a2 | B4b1 | B4c1b | B4c2 | B5a | B5b | C | D* | D4 |
| --- | --- | --- | --- | --- | --- | --- | --- | --- | --- | --- | --- | --- | --- |
| **Iban** |  |  | **13.58** |  |  |  | **3.70** | **4.94** |  |  |  |  |  |
| China Jorde |  |  |  |  |  |  |  |  |  |  |  | 30.77 |  |
| Japan Jorde |  |  |  |  |  |  |  |  |  |  |  | 60 |  |
| Malaysia Jorde |  |  |  |  |  |  |  |  |  |  |  |  |  |
| Taiwan Jorde |  |  |  |  |  |  |  |  |  |  |  |  |  |
| Vietnam Jorde |  |  |  |  |  |  |  |  |  |  |  |  |  |
| Cambodia Jorde |  |  |  |  |  |  |  |  |  |  |  |  |  |
| SE Asia Jorde |  |  |  |  |  |  |  |  |  |  |  |  |  |
| Melayu Malays |  |  | 0.9 |  |  | 0.9 | 2.7 | 2.8 | 9.2 | 0.9 | 0.9 |  |  |
| Taiwan |  | 0.4 | 9.2 |  | 11.5 | 6.6 | 3.3 |  | 5.1 |  |  | 0.2 | 0.9 |
| Phillipines |  |  | 11.3 |  |  | 1.6 |  |  |  | 9.7 |  |  |  |
| Sumatra | 3.9 |  | 6.1 |  |  | 0.6 | 4.4 | 2.2 | 3.9 | 2.2 |  |  |  |
| Borneo |  | 0.6 | 7 | 1.3 |  | 1.9 | 1.9 | 5.7 | 4.5 | 1.3 | 1.3 | 0.6 |  |
| Java | 2.2 |  | 2.2 |  |  |  | 2.2 |  | 2.2 |  | 2.2 |  |  |
| Bali |  | 1.2 | 2.4 |  |  |  | 6 | 7.2 | 4.8 |  |  |  |  |
| Lombok |  |  | 4.5 | 2.3 |  |  |  |  | 6.8 | 2.3 | 2.3 |  |  |
| Sumba |  |  | 2 |  |  |  |  |  |  |  |  |  |  |
| Sulawesi | 0.4 | 0.4 | 5.1 | 3.8 |  | 0.8 | 3.8 | 0.8 | 2.5 | 2.5 | 0.4 | 2.5 |  |
| Alor |  |  |  | 2.2 |  | 6.6 |  |  | 4.4 |  |  |  | 2.2 |
| Ambon |  |  | 9.3 | 14 |  | 4.7 |  |  | 7 |  |  | 4.7 |  |
| Total ISEA | 0.9 | 0.3 | 5.5 | 2 |  | 0.9 | 3 | 2.2 | 3.5 | 2 | 0.5 | 0.9 | 0.1 |
| NW China | 0.2 | 1.2 | 1 |  |  | 2 | 0.2 |  | 2.2 | 1 | 4.7 | 9.9 | 3.2 |
| NE China | 0.5 | 2.1 | 3.4 |  | 0.2 | 2.7 | 2.1 |  | 3.4 | 1.4 | 4.3 | 12.6 | 3.4 |
| SW China | 0.8 | 2.8 | 6.5 |  | 0.3 | 1.8 | 0.3 | 0.8 | 6.8 | 0.6 | 5.7 | 4.9 | 4.6 |
| SE China | 0.5 | 2 | 6.1 |  | 0.2 | 3.1 | 1.6 | 1 | 4.9 | 2.6 | 5.2 | 6.3 | 4.7 |
| Thailand | 0.4 | 3.7 | 5.3 |  |  |  |  | 2.3 | 10.5 |  | 3.6 | 4 | 0.4 |
| Orang Asli | 1.2 | 0.8 |  |  |  |  |  |  | 0.8 | 5.8 |  |  |  |

| D5 | E1* | E1a | E1b | E2 | F* | F1a* | F1a1* | F1a1a | F1a3 | F1a4 | F1a5 | F1b | F2 | F3a |
| --- | --- | --- | --- | --- | --- | --- | --- | --- | --- | --- | --- | --- | --- | --- |
|  | **1.23** |  |  | **1.23** |  |  |  | **3.70** |  | **1.23** |  |  |  |  |
| 0.9 |  | 0.9 | 3.7 | 0.9 |  | 3.8 | 3.7 | 8.3 | 1.9 |  |  |  |  | 0.9 |
| 4.1 | 2.4 | 6.2 |  | 3.1 |  | 0.2 | 2.5 |  | 0.7 | 0.7 |  |  | 0.5 |  |
|  |  | 8.1 |  | 1.6 |  | 1.6 |  |  | 8.2 |  |  |  |  |  |
|  | 0.6 | 3.3 | 2.2 | 0.6 |  | 4.4 |  | 5 | 1.1 | 1.1 | 1.7 |  |  |  |
| 4.5 | 0.6 | 8.9 | 1.9 | 3.2 | 2.5 | 0.6 | 1.3 | 0.6 | 1.9 | 1.9 |  |  |  |  |
|  |  |  | 2.2 |  |  | 2.8 | 2.2 | 4.4 | 5.6 |  | 13 |  |  |  |
| 1.2 | 1.2 | 3.6 | 1.2 |  | 1.5 | 6 | 4.8 | 2.4 |  |  |  |  |  |  |
| 2.3 |  | 2.3 | 6.8 |  | 2.3 | 9.1 |  | 9.1 | 2.2 | 2.3 |  |  |  |  |
|  |  | 2 | 12 | 2 |  | 2 | 2 | 4 | 4 | 6 |  |  |  |  |
| 8.4 | 2.1 | 17.7 | 3.8 | 2.9 |  | 1.7 | 0.4 | 1.3 | 1.7 | 5.1 | 0.4 | 0.4 |  |  |
|  |  | 6.6 | 4.4 |  |  | 2.2 |  | 2.2 |  | 6.7 |  |  |  |  |
| 2.3 | 7 | 4.7 | 0 | 4.7 |  |  |  | 2.3 | 7 | 4.7 |  |  |  |  |
| 3.2 |  | 8 | 3 |  | 0.6 | 2.8 | 0.9 | 2.6 | 2.3 | 2.9 | 1 | 0.1 |  |  |
| 3 | 0.2 |  |  |  | 0.7 | 1 | 2.5 | 0.7 |  |  |  | 2 | 1 | 0.7 |
| 7.1 |  |  |  |  | 1.4 | 2.1 | 2.5 | 0.2 | 0.2 | 0.2 |  | 2.7 | 1.8 | 0.9 |
| 2.6 | 0.1 |  |  |  | 0.6 | 4.9 | 1.9 | 3.7 |  | 0.2 |  | 2.8 | 2.9 | 1.1 |
| 5.6 | 0.2 |  |  |  | 2.4 | 2.9 | 3.3 | 2.7 |  | 0 |  | 3.3 | 1.9 | 1.4 |
| 1.6 |  |  |  |  |  | 4.7 | 1.2 | 8.9 |  | 0.5 |  | 0.8 | 2.4 | 1.6 |
|  |  |  |  |  |  |  |  | 10.9 |  |  |  |  |  |  |

| F3b | F4 | I | M* | M7* | M7b* | M7b1 | M7b3 | M7c1* | M7c1a | M7c1c | M12 | M21a | M21b | M21d |
| --- | --- | --- | --- | --- | --- | --- | --- | --- | --- | --- | --- | --- | --- | --- |
|  |  |  | **16.05** |  |  | **13.58** | **1.23** |  |  |  |  | **1.23** |  |  |
|  |  |  | 0 | 30.77 |  |  |  |  |  |  |  |  |  |  |
|  |  |  | 6.67 | 13.33 |  |  |  |  |  |  |  |  |  |  |
|  |  |  | 25 |  |  |  |  |  |  |  |  |  |  |  |
|  |  |  |  | 33.33 |  |  |  |  |  |  | 33.33 |  |  |  |
|  |  |  | 12.5 | 25 |  |  |  |  |  |  |  |  |  |  |
|  |  |  | 33.33 |  |  |  |  |  |  |  |  |  |  |  |
|  |  |  | 23.08 | 11.54 |  |  |  |  |  |  | 3.85 |  |  |  |
|  |  |  | 14.2 |  |  | 3.7 |  | 0.9 |  | 4.6 | 2.8 | 4.6 | 0.9 | 0.9 |
| 7.1 | 11.8 |  | 0.7 |  | 0.1 | 0.7 | 10.2 |  | 1.2 | 4.2 |  |  |  |  |
| 19 |  |  | 2.4 |  |  |  |  |  |  | 11.3 |  |  |  |  |
|  | 1.7 | 0.6 | 10.1 | 2.8 | 0.6 | 1.7 |  |  |  | 8.9 |  |  | 0.6 |  |
| 5.7 |  |  | 8.3 | 0.6 | 1.3 | 0.6 |  |  | 0.6 | 7 | 0.6 | 0.6 | 1.3 |  |
|  |  |  | 13 |  |  | 2.2 |  |  |  | 10.9 |  |  |  |  |
|  |  |  | 12 | 1.2 | 1.2 | 4.8 |  |  |  | 6 | 1.2 |  |  | 2.4 |
|  |  |  | 11.4 |  |  | 6.8 |  |  |  | 2.3 |  |  |  |  |
| 2 |  |  | 8 | 2 |  | 0 | 8 |  |  | 12 |  |  |  |  |
|  |  |  | 3 | 1.3 |  | 0.4 | 1.3 | 0.4 |  | 11 |  |  | 0.4 |  |
|  |  |  | 2.2 |  |  |  |  |  |  | 4.4 |  |  |  |  |
|  |  |  | 2.3 | 2.3 | 2.3 | 2.3 |  |  |  | 2.3 |  |  |  |  |
| 2.5 | 0.9 | 0.1 | 6.9 | 1.2 | 0.5 | 1.4 | 0.8 | 0.1 | 0.2 | 8.3 | 0.2 | 0.1 | 0.4 | 0.2 |
|  | 0.2 |  | 11.1 | 0.7 | 1 | 1.7 | 0.2 | 1.2 |  |  |  |  |  |  |
|  |  |  | 4.1 | 0.2 | 1.1 | 3.2 |  | 0.2 | 0.5 | 0.5 |  |  |  |  |
| 0.01 | 0.2 |  | 8 | 0.9 | 3.4 | 4.6 |  | 0.3 |  | 0.2 | 0.2 |  |  |  |
|  |  |  | 7 | 1.2 | 3 | 2.1 |  | 0.9 | 0.7 | 0 | 0.7 |  |  |  |
| 0.2 | 0.8 |  | 4.7 | 0.8 | 2 | 2.8 |  | 1.2 |  | 0.4 | 2.4 | 6.9 | 0.4 |  |
|  |  |  | 1.6 |  |  |  |  |  | 0.4 | 3.1 |  | 21 | 3.5 |  |

| M45 | M46 | M47 | N* | N9a6 | N21 | N22 | P | Q | R* | R9* | R9b | R9c | R22 | R23 |
| --- | --- | --- | --- | --- | --- | --- | --- | --- | --- | --- | --- | --- | --- | --- |
|  |  |  | **9.88** |  |  |  |  |  |  |  |  | **2.47** | **2.47** |  |
|  |  |  | 30.77 |  |  |  |  |  |  |  |  |  |  |  |
|  |  |  | 6.67 |  |  |  |  |  |  |  |  |  |  |  |
|  |  |  | 75 |  |  |  |  |  |  |  |  |  |  |  |
|  |  |  | 33.33 |  |  |  |  |  |  |  |  |  |  |  |
|  |  |  | 50 |  |  |  |  |  |  |  |  |  |  |  |
|  |  |  | 44.44 |  |  |  |  |  |  |  |  |  |  |  |
|  |  |  | 50 |  |  |  |  |  |  |  |  |  |  |  |
| 1.9 | 3.8 |  | 1.8 | 2.8 | 1.8 |  | 0.9 | 1.8 | 1.8 | 0.9 | 0.9 |  |  |  |
|  |  |  |  |  |  |  |  |  | 0 |  |  | 1.8 |  |  |
|  |  |  |  |  |  |  |  |  | 1.6 |  |  | 3.3 |  |  |
| 3.9 | 5 | 1.1 | 2.8 | 3.3 | 0.6 |  |  |  |  | 2.2 | 1.7 |  | 0.6 |  |
| 2.5 | 6.4 |  | 0.6 | 0.6 |  |  |  | 1.3 | 0.6 |  | 0.6 | 1.9 | 1.9 |  |
| 2.2 | 4.3 |  | 15.2 | 2.2 |  |  |  |  |  |  | 4.4 |  | 2.2 |  |
| 4.8 | 4.8 |  |  |  | 1.2 |  |  | 1.2 |  | 1.2 |  |  | 7.2 | 6 |
| 2.3 | 9.1 |  |  |  |  |  |  |  |  |  | 2.3 |  | 11.4 |  |
| 2 |  |  |  |  |  | 8 | 4 | 4 |  |  |  | 2 | 8 | 2 |
| 1.3 | 3 |  |  | 0.8 | 0.4 |  | 1.3 | 2.1 |  |  | 0.8 | 0.8 | 0.4 |  |
|  | 4.4 |  |  |  | 4.4 |  |  | 28.9 |  |  |  | 11.1 | 2.2 |  |
| 2.3 | 2.3 |  |  |  |  |  |  | 11.6 |  |  |  |  |  |  |
| 2.3 | 4 | 0.2 | 1.3 | 1.1 | 0.5 | 0.4 | 0.5 | 2.9 | 0.2 | 0.6 | 0.9 | 1.3 | 2.3 | 0.6 |
|  |  |  | 2 |  |  |  |  |  |  |  | 0.7 | 0.2 |  |  |
|  |  |  | 1.8 |  |  |  |  |  | 0.2 | 0.2 | 0.2 | 0.2 |  |  |
| 0.5 |  |  | 0.4 | 0.3 |  |  |  |  | 0.7 | 0.3 | 1.7 | 0.3 |  |  |
|  |  |  | 0.8 |  |  |  |  |  | 0.6 | 0 | 0.9 | 0.4 |  |  |
| 0.5 |  |  |  | 0.8 | 0.4 |  |  |  |  |  | 1.2 | 0.9 | 0.8 |  |
|  |  |  |  | 6.2 | 9.3 | 1.6 |  |  |  |  | 9.3 |  |  |  |

| U7 | Y2 | Z |
| --- | --- | --- |
|  | **12.35** | **11.11** |
|  |  | 7.69 |
|  |  | 6.67 |
|  |  |  |
|  |  |  |
|  |  | 12.5 |
|  |  | 22.22 |
|  |  | 11.54 |
|  | 1.8 |  |
|  | 1.2 |  |
|  | 12.9 |  |
| 0.6 | 6.7 | 0.6 |
|  | 1.9 | 0.3 |
|  | 2.2 |  |
|  | 1.2 |  |
|  |  |  |
|  |  |  |
|  | 1.7 |  |
|  |  |  |
|  |  |  |
| 0.1 | 2.9 | 0.4 |
|  |  | 3.2 |
|  | 0.7 | 4.6 |
|  |  | 1.3 |
|  |  | 1.2 |
|  |  | 0.4 |
|  |  |  |
