# Supplementary material for: Ancestry of the Iban Is Predominantly Southeast Asian: Genetic Evidence from Autosomal, Mitochondrial, and Y Chromosomes
Source: PLoS One. 2011 Jan 31;6(1):e16338. doi: 10.1371/journal.pone.0016338 (PMC3031551; doi:10.1371/journal.pone.0016338)
Supplement: Table S5 — MtDNA haplogroup definitions for the Iban population. (DOCX) [file pone.0016338.s006.docx]

Table S5. MtDNA haplogroup definitions for the Iban population

| **Sample ID** | **Derived SNP states used to determine haplotypes** | | | | | | | | | | | | **Haplogroup** | |
| --- | --- | --- | --- | --- | --- | --- | --- | --- | --- | --- | --- | --- | --- | --- |
| 1 | 8701 | 10398 | 15301 | 10873 | 16189 | 16217 | 16261 |  |  |  |  |  | | B4a |
| 2 | 8701 | 10398 | 15301 | 10873 | 16189 | 16217 | 16261 |  |  |  |  |  | | B4a |
| 3 | 8701 | 10398 | 10873 | 16189 | 16217 | 16261 |  |  |  |  |  |  | | B4a |
| 4 | 8701 | 10398 | 15301 | 10873 | 16189 | 16217 | 16261 |  |  |  |  |  | | B4a |
| 5 | 8701 | 10398 | 15301 | 10873 | 16189 | 16217 | 16261 |  |  |  |  |  | | B4a |
| 6 | 8701 | 10398 | 15301 | 10873 | 16189 | 16217 | 16261 |  |  |  |  |  | | B4a |
| 7 | 8701 | 10398 | 15301 | 10873 | 16189 | 16217 | 16261 |  |  |  |  |  | | B4a |
| 8 | 8701 | 10398 | 15301 | 10873 | 16129 | 16189 | 16217 | 16261 |  |  |  |  | | B4a |
| 9 | 8701 | 10398 | 10873 | 16189 | 16217 | 16261 |  |  |  |  |  |  | | B4a |
| 10 | 8701 | 10398 | 15301 | 10873 | 16189 | 16217 | 16261 |  |  |  |  |  | | B4a |
| 11 | 8701 | 10398 | 15301 | 10873 | 16129 | 16189 | 16217 | 16261 |  |  |  |  | | B4a |
| 12 | 8701 | 10398 | 10873 | 16140 | 16189 | 16217 | 16274 | 16335 |  |  |  |  | | B4c1b3 |
| 13 | 8701 | 10398 | 10873 | 16140 | 16189 | 16217 | 16223 | 16274 | 16335 |  |  |  | | B4c1b3 |
| 14 | 8701 | 10398 | 10873 | 16140 | 16189 | 16217 | 16274 | 16335 |  |  |  |  | | B4c1b3 |
| 15 | 8701 | 10398 | 15301 | 10873 | 16147 | 16184 | 16189 | 16217 | 16235 |  |  |  | | B4c2 |
| 16 | 8701 | 10398 | 15301 | 10873 | 16147 | 16184 | 16189 | 16217 | 16235 |  |  |  | | B4c2 |
| 17 | 8701 | 10398 | 10873 | 16147 | 16184 | 16189 | 16217 | 16235 |  |  |  |  | | B4c2 |
| 18 | 8701 | 10398 | 15301 | 10873 | 16147 | 16184 | 16189 | 16217 | 16235 |  |  |  | | B4c2 |
| 19 | 489 | 15043 | 4491 | 7598 | 16223 | 16362 | 16390 |  |  |  |  |  | | E1 |
| 20 | 489 | 15043 | 4491 | 7598 | 16051 | 16223 | 16362 | 16390 |  |  |  |  | | E2 |
| 21 | 8701 | 10398 | 15301 | 10873 | 16108 | 16129 | 16162 | 16172 | 16304 |  |  |  | | F1a1a |
| 22 | 8701 | 10398 | 15301 | 10873 | 16108 | 16129 | 16162 | 16172 | 16304 |  |  |  | | F1a1a |
| 23 | 8701 | 10398 | 15301 | 10873 | 16108 | 16129 | 16162 | 16172 | 16304 |  |  |  | | F1a1a |
| 24 | 8701 | 10398 | 15301 | 10873 | 16172 | 16249 | 16288 | 16301 | 16304 | 16362 | 16390 |  | | F1a4 |
| 25 | 489 | 15043 | 16223 | 16311 |  |  |  |  |  |  |  |  | | M* |
| 26 | 16157 | 16256 | 16304 | 16335 |  |  |  |  |  |  |  |  | | M* |
| 27 | 489 | 15043 | 16223 |  |  |  |  |  |  |  |  |  | | M* |
| 28 | 489 | 15043 | 16223 | 16311 | 16362 |  |  |  |  |  |  |  | | M* |
| 29 | 489 | 15043 | 152 | 16223 | 16311 |  |  |  |  |  |  |  | | M* |
| 30 | 489 | 15043 | 16148 | 16223 | 16261 |  |  |  |  |  |  |  | | M* |
| 31 | 489 | 15043 | 16093 | 16148 | 16223 |  |  |  |  |  |  |  | | M* |
| 32 | 489 | 15043 | 16148 | 16223 | 16261 |  |  |  |  |  |  |  | | M* |
| 33 | 489 | 15043 | 16223 |  |  |  |  |  |  |  |  |  | | M* |
| 34 | 489 | 15043 | 16093 | 16148 | 16223 |  |  |  |  |  |  |  | | M* |
| 35 | 489 | 15043 | 16148 | 16223 | 16261 |  |  |  |  |  |  |  | | M* |
| 36 | 489 | 15043 | 16148 | 16223 |  |  |  |  |  |  |  |  | | M* |
| 37 | 489 | 15043 | 16223 | 16311 | 16362 |  |  |  |  |  |  |  | | M* |
| 38 | 489 | 15043 | 11482 | 16223 |  |  |  |  |  |  |  |  | | M21 |
| 39 | 489 | 15043 | 6455 | 199 | 16129 | 16189 | 16192 | 16223 | 16294 | 16297 |  |  | | M7b1 |
| 40 | 489 | 15043 | 6455 | 199 | 16129 | 16189 | 16192 | 16223 | 16294 | 16297 |  |  | | M7b1 |
| 41 | 489 | 15043 | 6455 | 199 | 16129 | 16189 | 16192 | 16223 | 16294 | 16297 |  |  | | M7b1 |
| 42 | 489 | 15043 | 6455 | 199 | 16129 | 16189 | 16192 | 16223 | 16294 | 16297 |  |  | | M7b1 |
| 43 | 489 | 15043 | 6455 | 16129 | 16189 | 16192 | 16223 | 16294 | 16297 |  |  |  | | M7b1 |
| 44 | 489 | 15043 | 6455 | 199 | 16129 | 16189 | 16193 | 16223 | 16294 | 16297 |  |  | | M7b1 |
| 45 | 489 | 15043 | 6455 | 199 | 16129 | 16189 | 16193 | 16223 | 16294 | 16297 |  |  | | M7b1 |
| 46 | 489 | 15043 | 6455 | 199 | 16129 | 16189 | 16192 | 16223 | 16294 | 16297 |  |  | | M7b1 |
| 47 | 489 | 15043 | 6455 | 199 | 16129 | 16189 | 16192 | 16223 | 16294 | 16297 |  |  | | M7b1 |
| 48 | 489 | 15043 | 6455 | 16189 | 16193 | 16223 | 16294 | 16297 |  |  |  |  | | M7b1 |
| 49 | 489 | 15043 | 6455 | 199 | 16129 | 16189 | 16192 | 16223 | 16294 | 16297 |  |  | | M7b1 |
| 50 | 489 | 15043 | 6455 | 199 | 16223 | 16311 |  |  |  |  |  |  | | M7bc |
| 51 | 489 | 15043 | 6455 | 199 | 16126 | 16231 | 16311 |  |  |  |  |  | | M7bc |
| 52 | 489 | 15043 | 6455 | 199 | 16126 | 16223 | 16297 |  |  |  |  |  | | M7b3 |
| 53 | 8701 | 15301 | 10873 | 16189 | 16266 |  |  |  |  |  |  |  | | N |
| 54 | 8701 | 10398 | 15301 | 10873 | 16274 | 16298 | 16311 |  |  |  |  |  | | N |
| 55 | 8701 | 10398 | 15301 | 10873 | 16189 | 16217 | 16261 |  |  |  |  |  | | N |
| 56 | 8701 | 10398 | 15301 | 10873 | 16140 | 16189 | 16266 |  |  |  |  |  | | N |
| 57 | 8701 | 15301 | 10873 | 16129 | 16172 | 16193 | 16304 |  |  |  |  |  | | N |
| 58 | 8701 | 10398 | 15301 | 10873 | 16129 | 16189 | 16193 |  |  |  |  |  | | N |
| 59 | 8701 | 10398 | 15301 | 10873 | 16129 | 16172 | 16193 | 16304 |  |  |  |  | | N |
| 60 | 8701 | 15301 | 10873 | 16140 | 16189 | 16266 |  |  |  |  |  |  | | N |
| 61 | 8701 | 10398 | 15301 | 10873 | 16172 | 16249 | 16288 | 16301 | 16304 | 16362 | 16390 |  | | R22 |
| 62 | 8701 | 10398 | 15301 | 10873 | 16249 | 16288 | 16304 | 16335 |  |  |  |  | | R22 |
| 63 | 8701 | 10398 | 15301 | 10873 | 16157 | 16256 | 16304 | 16335 |  |  |  |  | | R9c |
| 64 | 8701 | 10398 | 15301 | 10873 | 16157 | 16256 | 16304 | 16335 |  |  |  |  | | R9c |
| 65 | 8701 | 15301 | 10873 | 16274 | 16298 | 16311 | 16362 |  |  |  |  |  | | Y2 |
| 66 | 8701 | 10398 | 15301 | 10873 | 16274 | 16298 | 16311 | 16362 |  |  |  |  | | Y2 |
| 67 | 8701 | 15301 | 10873 | 5417 | 16126 | 16231 | 16311 |  |  |  |  |  | | Y2 |
| 68 | 8701 | 15301 | 10873 | 5417 | 16223 | 16311 |  |  |  |  |  |  | | Y2 |
| 69 | 8701 | 10398 | 15301 | 10873 | 16223 | 16311 |  |  |  |  |  |  | | Y2 |
| 70 | 8701 | 15301 | 10873 | 5417 | 16126 | 16231 | 16311 |  |  |  |  |  | | Y2 |
| 71 | 8701 | 10398 | 15301 | 10873 | 16274 | 16298 | 16311 | 16362 |  |  |  |  | | Y2 |
| 72 | 8701 | 15301 | 10873 | 5417 | 16126 | 16231 | 16311 |  |  |  |  |  | | Y2 |
| 73 | 8701 | 15301 | 10873 | 5417 | 16126 | 16231 | 16311 |  |  |  |  |  | | Y2 |
| 74 | 8701 | 15301 | 10873 | 5417 |  |  |  |  |  |  |  |  | | Y2 |
| 75 | 489 | 15043 | 152 | 16223 | 16311 |  |  |  |  |  |  |  | | Z |
| 76 | 489 | 15043 | 152 | 16129 | 16209 | 16223 |  |  |  |  |  |  | | Z |
| 77 | 489 | 15043 | 152 | 16223 | 16311 |  |  |  |  |  |  |  | | Z |
| 78 | 489 | 15043 | 152 | 16129 | 16189 | 16192 | 16223 | 16294 | 16297 |  |  |  | | Z |
| 79 | 489 | 15043 | 152 | 16189 | 16217 | 16261 |  |  |  |  |  |  | | Z |
| 80 | 489 | 15043 | 152 | 16189 | 16217 | 16232 | 16233 | 16235 | 16261 | 16390 |  |  | | Z |
| 81 | 489 | 15043 | 152 | 16223 | 16311 |  |  |  |  |  |  |  | | Z |
| 82 | 489 | 15043 | 152 | 16129 | 16209 | 16223 |  |  |  |  |  |  | | Z |
| 83 | 489 | 15043 | 152 | 16223 | 16311 |  |  |  |  |  |  |  | | Z |
