# Supplementary material for: Ancestry of the Iban Is Predominantly Southeast Asian: Genetic Evidence from Autosomal, Mitochondrial, and Y Chromosomes
Source: PLoS One. 2011 Jan 31;6(1):e16338. doi: 10.1371/journal.pone.0016338 (PMC3031551; doi:10.1371/journal.pone.0016338)
Supplement: Table S6 — SNP heterozygosity. (DOCX) [file pone.0016338.s007.docx]

Table S6. SNP heterozygosity

| CHB | 0.2883 |
| --- | --- |
| Chinese | 0.287 |
| Iban | 0.285 |
| Indonesia | 0.2887 |
| Japanese | 0.3008 |
| JPT | 0.2874 |
| Khmer Cambodian | 0.2968 |
| Malaysia | 0.2852 |
| Philippines | 0.2778 |
| Taiwan | 0.27 |
| Thailand | 0.2802 |
| Vietnamese | 0.2893 |
