# Supplementary material for: Ancestry of the Iban Is Predominantly Southeast Asian: Genetic Evidence from Autosomal, Mitochondrial, and Y Chromosomes
Source: PLoS One. 2011 Jan 31;6(1):e16338. doi: 10.1371/journal.pone.0016338 (PMC3031551; doi:10.1371/journal.pone.0016338)
Supplement: Table S7 — RSTestimates based on STR analysis. (DOCX) [file pone.0016338.s008.docx]

Table S7. *R_ST_* estimates based on STR analysis

| **Population** | Cambodian | Chinese | Japanese | Malaysian | Vietnamese | Iban |
| --- | --- | --- | --- | --- | --- | --- |
| Cambodian | - |  |  |  |  |  |
| Chinese | 0.0000 | - |  |  |  |  |
| Japanese | 0.0000 | 0.0137 | - |  |  |  |
| Malaysian | 0.0000 | 0.0000 | 0.0193 | - |  |  |
| Vietnamese | 0.0305 | 0.0122 | 0.0104 | 0.0000 | - |  |
| Iban | 0.0301 | 0.0454 | 0.0344 | 0.0083 | 0.0264 | - |
