# Supplementary material for: Ancestry of the Iban Is Predominantly Southeast Asian: Genetic Evidence from Autosomal, Mitochondrial, and Y Chromosomes
Source: PLoS One. 2011 Jan 31;6(1):e16338. doi: 10.1371/journal.pone.0016338 (PMC3031551; doi:10.1371/journal.pone.0016338)
Supplement: Table S8 — Nucleotide diversity estimates for HVS-1 mtDNA. (DOCX) [file pone.0016338.s009.docx]

Table S8. Nucleotide diversity estimates for HVS-1 mtDNA

| **Population** | **N** | **Nucleotide diversity** |
| --- | --- | --- |
| Africans | 143 | 0.025 |
| Iban | 81 | 0.019 |
| South Indians | 114 | 0.016 |
| Southeast Asians | 27 | 0.015 |
| East Asians | 36 | 0.014 |
| Central Asians | 145 | 0.014 |
| Europeans | 119 | 0.008 |
